# A Comparative Study of Genetic Diversity and Multiplicity of Infection in Uncomplicated *Plasmodium falciparum* Infections in Selected Regions of Pre-Elimination and High Transmission Settings Using *MSP1* and *MSP2* Genes

**DOI:** 10.3390/pathogens13020172

**Published:** 2024-02-13

**Authors:** Olusegun Philip Akoniyon, Moses Akiibinu, Matthew A. Adeleke, Rajendra Maharaj, Moses Okpeku

**Affiliations:** 1 Discipline of Genetics, School of Life Sciences, University of KwaZulu-Natal, Westville Campus, Durban 4041, South Africa; akohseg@gmail.com (O.P.A.); adelekem@ukzn.ac.za (M.A.A.); 2 Department of Biochemistry and Chemistry, Caleb University, Lagos 11379, Nigeria; akiibinumoses1@gmail.com; 3 Office of Malaria Research, South African Medical Research Council, Cape Town 7505, South Africa; rmaharaj@mrc.ac.za

**Keywords:** *P. falciparum*, *msp1*, *msp2*, MOI, South Africa, Nigeria

## Abstract

Background: Understanding the genetic structure of *P*. *falciparum* population in different regions is pivotal to malaria elimination. Genetic diversity and the multiplicity of infection are indicators used for measuring malaria endemicity across different transmission settings. Therefore, this study characterized *P. falciparum* infections from selected areas constituting pre-elimination and high transmission settings in South Africa and Nigeria, respectively. Methods: Parasite genomic DNA was extracted from 129 participants with uncomplicated *P. falciparum* infections. Isolates were collected from 78 participants in South Africa (southern Africa) and 51 in Nigeria (western Africa). Allelic typing of the *msp1* and *msp2* genes was carried out using nested PCR. Results: In *msp1*, the K1 allele (39.7%) was the most common allele among the South African isolates, while the RO33 allele (90.2%) was the most common allele among the Nigerian isolates. In the *msp2* gene, FC27 and IC3D7 showed almost the same percentage distribution (44.9% and 43.6%) in the South African isolates, whereas FC27 had the highest percentage distribution (60.8%) in the Nigerian isolates. The *msp2* gene showed highly distinctive genotypes, indicating high genetic diversity in the South African isolates, whereas *msp1* showed high genetic diversity in the Nigerian isolates. The RO33 allelic family displayed an inverse relationship with participants’ age in the Nigerian isolates. The overall multiplicity of infection (MOI) was significantly higher in Nigeria (2.87) than in South Africa (2.44) (*p* < 0.000 *). In addition, heterozygosity was moderately higher in South Africa (1.46) than in Nigeria (1.13). Conclusions: The high genetic diversity and MOI in *P. falciparum* that were observed in this study could provide surveillance data, on the basis of which appropriate control strategies should be adopted.

## 1. Introduction

Malaria, a parasitic disease caused by infection with the protozoan parasite species of the genus *Plasmodium*, still wreaks havoc in the general health of human populations in the tropical and sub-tropical regions of the world [1]. Prior to the COVID-19 pandemic, global malaria morbidity and mortality witnessed a significant reduction. In 2018, 228 million cases were reported globally, as against 231 million cases in 2017 and 251 million cases in 2010, causing 405,000 deaths in 2018, as against 416,000 deaths in 2017 globally [2]. However, the World Health Organization (WHO) recently reported 247 million malaria cases in 2021, as against 245 million cases reported in 2020 and 231 million cases in 2019. This marked increase might be partly due to the COVID-19 pandemic, which caused interruptions of malaria control interventions in many malaria-endemic countries [3], as funds for malaria control were diverted to curtail the effects of the pandemic in low and middle income countries [4,5]. WHO estimates indicate that African regions have the largest malaria burden in the world, accounting for 95% of cases and 96% of deaths from the disease, with children under the age of five accounting for 80% of these deaths [3].

Malaria, predominantly caused by *P. falciparum* in African regions, continues to affect all age groups and is disproportionately distributed across the region [2]. South Africa is at the pre-elimination phase, with three of its provinces showing different levels of endemicity [6]. In KwaZulu-Natal (KZN), for example, there was an increase of 124 in the number of local malaria cases and 5 deaths in 2018 [7]. A similar study substantiated this report, whereby a significant reduction in malaria cases from 1459 to 70 was observed between 2018 and 2019 [8]. When compared to the other two malaria-endemic provinces in South Africa, the Vhembe District Municipality had the highest malaria morbidity in 2017–2018 transmission season [9], whereas malaria in Nigeria is perennial and contributes the highest malaria burden globally, with 97% of the population at risk of infection with malaria [10]. Ibadan in the southwestern part of Nigeria, where the study was conducted, contributed 16% to the national malaria prevalence in 2021 [11], and malaria incidence rates in the six geopolitical zones showed that South West Nigeria has the lowest incidence of cases after the south east, with the northern regions bearing the lion’s share [12]. 

The goal of eradicating malaria has been hampered by factors like the evolution and spread of drug-resistant *P. falciparum* strains and the ineffectiveness of malaria vaccines [13,14]. The high malaria burden in SSA requires the constant surveillance of parasite diversity that enables the parasites to develop resistance to antimalarials, thereby surviving control measures such as antimalarial treatment [15]. There is a relationship between genetic diversity and multiplicity of infection (MOI). Low transmission settings exhibit low genetic diversity, while regions with high transmission show high genetic diversity [16]. In addition, factors such as the size of the effective population, the rate of mutation at the loci being studied and the genotyping technique make genetic diversity difficult to estimate [17]. Also, there are spatial genetic differences that seem contradictory, necessitating ongoing evaluation of the structure and diversity of *P. falciparum* in Africa [15]. The clonality of a region reflects the transmission intensity of the area and as such, influences the immune system of people residing in the area [18]. Thus, the characterization of parasite dynamics in low and high transmission settings is needed to decipher the implications of disease control strategies and to understand the parasite indices that mitigate different control strategies. 

Over time, evolution has occurred between *P. falciparum* and the human immune system to ensure that the parasites avoid elimination and establish reinfection after splenic clearance [19]. *Plasmodium* infection of red blood cells requires the expression of merozoite proteins. Understanding the molecular mechanisms of merozoite invasion and host immune responses to merozoite antigens is therefore critical for developing malaria vaccines and new treatments [20,21]. *Msp1* and *msp2* are two polymorphic proteins that have been extensively used in characterizing malaria parasites [22]. *Msp1*, with a 190–200 kDa protein, has undergone extensive research as a potential vaccine candidate. It is also the most prevalent protein on the surface of merozoites [23,24]. *Msp2* is a 25 kDa protein that is conspicuous on the merozoite surface and has been assumed to be implicated in erythrocyte invasion and parasite growth [21]. The *msp1* gene, located on chromosome 9, contains 17 blocks of sequences, of which block 2 is the most polymorphic and is grouped into three allelic families, K1, MAD20 and RO33 [25]. The *msp2* gene is located on chromosome 2 [26] comprising 5 blocks, where block 3 is the most polymorphic and is grouped into two allelic families, FC27 and IC1/3D7) [27]. The *msp1* and *msp2* genes are highly polymorphic and are thus useful in studying the *P. falciparum* population structure [28]. The MOI can be determined by genotyping infections with polymorphic markers such as merozoite surface protein-1 (*msp1*) and glutamate-rich protein [29]. This study aimed to compare the genetic diversity and multiplicity of infection in northern KwaZulu-Natal (KZN) and Limpopo, South Africa, representing a low transmission setting, and Ibadan, Nigeria, representing a high transmission setting, using the polymorphic *msp1* and *msp2* genes.

## 2. Materials and Methods

### 2.1. Ethical Clearance

Clearance for the Nigerian sample collection was obtained from the Ministry of Health, Oyo State, Nigeria (NHREC/OYOSHRIEC/10/11/22) and for the South African samples was obtained from the University of KwaZulu-Natal ethical committee (Biomedical Research Ethics Committee-BREC/00002766/2021). All participants were given consent forms to fill in confirming their voluntary interest in the study participation.

### 2.2. Study Sites

This work was carried out in northern KwaZulu-Natal and Limpopo, South Africa, representing a pre-elimination (5 cases of *P. falciparum* in 2022) setting, and in Ibadan, Oyo East, Nigeria, representing a high transmission setting. Sampling in South Africa was carried out in northern KwaZulu-Natal (KZN) (Jozini and Manguzi) and Limpopo (Mopani District), while sampling was carried out at a general hospital in Apata, Ibadan, Oyo State, Nigeria. The data presented here are based on preliminary samples collected in the course of the study. The village of Manguzi, which borders Mozambique, is located in KZN and has a high rate of sickness. Manguzi and Jozini are under the local municipality of uMhlabuyalingana. In total, 99% of the municipality’s geographical area—roughly 3621 km^2^—is devoted to rural settlements, hence the name “rural municipality”. Reserved areas and commercial farmlands make up 40% of the total land area. Situated in the northern region of KZN, the Jozini local municipality is bordered to the north by Mozambique, to the west by Swaziland, to the east by uMhlabuyalingana, to the south by Hlabisa, and to the west by Nongoma and uPhongolo. The Jozini local municipality makes up 3057 m^3^, or 32%, of the total land area. Manguzi and Jozini have a KZN subtropical climate with dry and cold winters spanning the April to August period, and hot and humid summers from September to March (the primary rainy season). Sample collection spanned the year.

In Nigeria, samples were collected in the Atiba Local Government Area, Ibadan, Oyo State. Ibadan is located in South West Nigeria and situated between latitude 7°21′2.48″ N and longitude 3°51′55.84″ E [30]. Located in a tropical region, its climatic conditions are formed by rainy and dry seasons. Farming is the prominent occupation in the rural centers, with thick grasslands creating a conducive environment for vector proliferation. The average annual temperature is around 27 °C, and there is approximately 2100 mm of rainfall [31]. Malaria transmission is perennial and the main parasite infection in this region is *P. falciparum* [32]. Samples were collected during the rainy season, when transmission peaks.

### 2.3. Study Design and Participants Inclusion

A cross-sectional survey design was adopted in this research. The blood samples from participants were spotted onto FTA cards Whatman 3MM™ (GE Healthcare, United Kingdom) using a new syringe for each individual. Venous blood was spotted onto WhatmanTM 3MM filter cards, air-dried at room temperature and then sealed in ziplock plastics containing silica gel to prevent moisture and DNA degradation. Participants who presented with uncomplicated malaria symptoms according to the WHO [3], which include fever (axillary temperature ≥ 37.5 °C), cephalgias, fatigue, malaise and anaemia, were included. Those who showed symptoms of severe malaria according to the WHO and pregnant women were excluded from the study. A well-structured questionnaire was used to collect the demographic and clinical data for each participant.

### 2.4. Microscopy

Blood films were prepared and examined under a microscope to confirm infection and determine the parasite density. Thick blood smears were prepared and stained with 10% fresh Giemsa, and parasite density was estimated by counting the number of asexual parasites against 200 leukocytes, assuming a leukocyte count of 8000 white cells/µL [15]. Parasite density was further categorized as light (<500), moderate (500–4999) and heavy (>5000 parasites/μL) [1]. 

### 2.5. Parasite Genomic DNA Extraction

Parasite DNA from the FTA cards was extracted using a QIAGEN DNeasy Blood and Tissue Kit (Qiagen, Hilden, Germany) according to the manufacturer’s instructions, as previously described, with slight modifications [33]. Eluates were stored at −20 °C for further usage. 

### 2.6. Confirmation of P. falciparum

*P. falciparum* confirmation was conducted by targeting the 18S rRNA of *P. falciparum* isolates, as previously described by Snonou et al. [34]. Briefly, the small sub-unit ribosomal RNA (ssrRNA) was amplified using primary (genus-specific) and nested (species-specific) primers for *P. falciparum* with little modification. The genomic DNA of a laboratory strain (3D7 strain) was used for positive control. Template-free (negative) control was incorporated into the research to check contamination. Isolates with *P. falciparum* monoinfections were included for allelic typing.

### 2.7. Typing of Msp1 and Msp2 Alleles

Allelic typing was conducted using the conserved polymorphic regions of *msp1* (block 2) and *msp2* (block 3) of *P. falciparum* according to a modified protocol previously described [35]. Primers in the primary reaction spanned the *msp1* and *msp2* loci, while the nested primers spanned the allele loci *msp1* (K1, MAD20 and RO33) and *msp2* (IC3D7 and FC27). All reactions were conducted in a final volume of 40 μL containing 2 μL of gDNA, 20 μL of DreamTaq Green Master Mix (Thermo Fisher Scientific, Waltham, MA, USA), 2 μL (1.0 μM) of forward and reverse primers and 14 μL of nuclease-free water. In both the primary and secondary reactions, 2 μL of gDNA and PCR amplicons were used, respectively, as templates for the primary and secondary PCR amplifications. The PCR amplification cycling conditions of the *msp* genes and the alleles included an initial denaturation of 95 °C for 3 min, a final denaturation of 95 °C for 30 s at 35 cycles with initial extension of 72 °C for 1 min and a final extension of 72 °C for 5 min with different annealing temperatures, as listed in Table 1. The primary and nested PCR assays were run separately for each gene and allele. The genomic DNA of cloned laboratory strains was employed as a positive control for the appropriate alleles, and a template-free control was used in each process. The primer sequences for *msp1* and *msp2* are listed in Table 1 [36]. The PCR products were analyzed using electrophoresis stained with ethidium bromide in 1.5% agarose gel to resolve the presence of alleles. DNA sizes were determined by a 100–1500 bp molecular weight marker and viewed using a UV image Gel Doc XR+ Imaging System (BioRad, Hercules, CA, USA). 

### 2.8. Statistical Analysis

Comparison of the allele proportion between countries was calculated using a Chi-squared test. The MOI is an index of malaria transmission intensity calculated by dividing the total number of alleles detected for *msp1* and *msp2* alleles by the total number of samples. An independent t-test was used to compare the MOI between countries [1]. Single infections were those having one allele in each locus genotyped, while multiclonal infections had more than one allele per locus [37]. *p*-values were significant if less than 0.05.

## 3. Results

### 3.1. General Characteristics

Out of 156 participants (84 from South Africa and 72 from Nigeria) recruited for this study, only 78 and 51 participants were included in the research in South Africa and Nigeria, respectively. Twenty-one of the Nigerian isolates and six South African isolates were excluded due to mixed infections or severe malaria symptoms. Because uncomplicated malaria is the prevalent type and is a precursor to other malaria types, it remains the most-studied infection. Among the participants, 26 (33.3%) were male and 52 (66.7%) were female in South Africa, while 18 (35.3%) were males and 33 (64.7%) were female in Nigeria. There was a significant difference in the mean age of the participants, with 32.41 (12.06%) and 20.73 (18.36%) in South Africa and Nigeria, respectively. 

### 3.2. Allelic Proportion of Msp1 and Msp2 Genes in South Africa and Nigeria

In *msp1*, the RO33 allele was the most prevalent allele (90.2%) among the monoclonal infections in the Nigerian isolates, whereas the K1 allele (39.7%) was the predominant type in the South African isolates. The K1 allele was more prevalent in females than in males. MAD20 + RO33, RO33 + K1, RO33 + MAD20 + K1 combinations were absent in males in the South African isolates but present in males in the Nigerian isolates. The percentage of the FC27 allele (44.9%) was close to that of the IC3D7 allele (43.6%) in the South African isolates, whereas FC27 allele was higher than IC3D7 allele with 60.8% and 19.6%, respectively, in the Nigerian isolates. The summary of the allelic proportions is presented in Table 2.

### 3.3. Allelic Diversity in Msp1 and Msp2 of P. falciparum in South African and Nigerian Isolates

**Figure 1:**
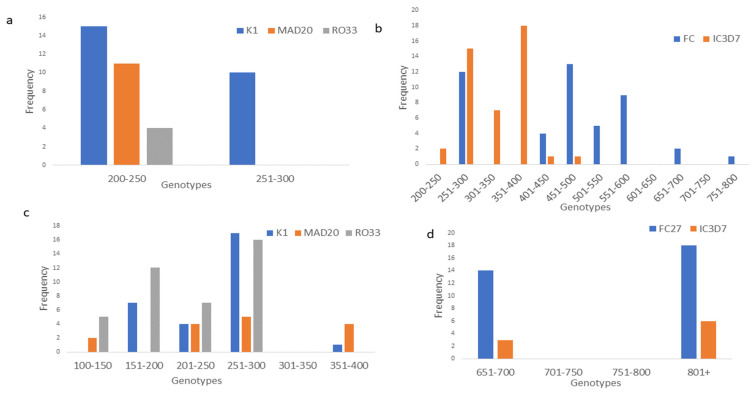
Genetic diversity in *msp1* (**a**) and *msp2* (**b**) in South African isolates; and in *msp1* (**c**) and *msp2* (**d**) in Nigerian isolates.

For the *msp1* gene analysis in South Africa, a total of 40 alleles were detected, comprising 25 K1 alleles with a size range of 200–300 bp, 11 MAD20 with 200 bp and 4 RO33 allele with size 200 bp. The *msp1* gene analysis in Nigeria showed a significant predominance of 40 RO33 alleles with a size range of 100–300 bp, followed by K1 with 29 alleles ranging from 200 to 400 bp and 15 MAD20 alleles with band size of 100–400 bp. In South Africa, 44 IC3D7 alleles were found with size ranges of 300–500 bp and 46 FC27 ranging from 300 to 800 bp, whereas in Nigeria, 32 FC27 alleles ranging from 700–900 bp were present in the isolates, while 09 IC3D7 alleles (700–900 bp) were detected in the *msp2* (Supplementary Figure S1). As shown in Table 3, the age of the participants was categorized into three groups: under 19; 20–40; and above 40. There was a significant inverse relationship between the age categories and the RO33 allele, as an increase in age leads to a decrease in the allele proportion, whether in the form of monoallelic or polyallelic infections. In *msp2*, FC27 was significantly distributed among the age categories. No significant relationship was observed between alleles and gender. The genetic diversity of *msp1* and *msp2* genes are represented in Figure 1.

Due to the similarity in the parasite densities of the participants, the parasite densities of the participants from the two countries were grouped into the following three categories: light (<500 parasites/µL); moderate (500–4999 parasites/µL) and heavy (>5000 parasites/µL) to see the relationship between the MOI and age. 

### 3.4. Multiplicity of Infection and Expected Heterozygosity (He)

Multiplicity of infection, defined as the number of clones present in an infection, showed a significant difference between South Africa and Nigeria in *msp1*, while no significant difference was observed in *msp2* between the countries. The overall multiplicity of infection showed a significant increase in the Nigerian isolates compared to the south African isolates, as depicted in Table 4. Heterozygosity was calculated using He = [n/(n − 1)] [(1 − ΣPi2)], where n = sample size and Pi = allele frequency, as previously described [1]. Heterozygosity in the *msp1* locus was higher in South Africa (0.81) compared to Nigeria (0.54) while the *msp2* findings were very similar between the countries, at 0.61 and 0.59, respectively.

## 4. Discussion

In order to curtail malaria transmission, robust strategic control measures that not only assess previous control interventions but also accurately inform appropriate methods of interrupting transmission within a geographical setting and preventing imported cases that can sustain residual transmission are required. The goal of malaria elimination in the present era will remain a far-fetched goal if the continuous monitoring of important malaria indices like transmission level, *Plasmodium* genetic diversity, drug resistance in parasites and insecticide resistance in the mosquito vector is not conducted well. Hence, this study aimed at a comparative study of genetic diversity in *P. falciparum msp1* and *msp2* genes from individuals infected with malaria in two different geographically placed countries (South Africa and Nigeria) with significant differences in malaria endemicity. The MOI and allelic diversity in polymorphic blocks of *msp1* and *msp2* were also assessed. 

This study observed a higher MOI in the *P. falciparum msp1* gene in the Nigerian isolates compared to the South African isolates. There was a significant discrepancy in the MOI of *msp1* between the countries, while *msp2* also displayed a similar difference, though not as significant (*p* = 0.213). The overall MOI was higher in the Nigerian isolates than in the South African isolates. This increase could partly contribute to clinical malaria in Nigeria [38]. Moreover, the high genetic diversity observed in the *msp1* Nigerian isolates in our study was also reported in another study [1]. The high diversity of *msp2* observed in our study was consistent with another study [39]. The high allelic proportion of IC3D7 observed in our study in South Africa was also reported in the Republic of the Congo [40] and Benin [41]. Among the *msp1* alleles, the K1 allele family was predominant in the South African isolates. This is consistent with what has been reported in Benin [42], Pakistan [43] and in other literature [1,37,44]. Our study showed that the allelic proportions of FC27 and IC3D7 were almost equal in the *msp2* allele family in South Africa. This result contradicts a study in Madagascar, where FC27 was the frequent allele family detected [45]. This dichotomy could result from a difference in the *Plasmodium* population structure, which depends on local epidemiological and demographic factors such as transmission intensity, migration of asymptomatic carriers, etc. [46].

The prevalence of the RO33 allele in our findings in Nigeria was consistent with another study conducted in Nigeria where RO33 was the dominant allele and the K1 family was the least occurring allele [15]. Our study also showed RO33 as the most frequent polyclonal infection, which is in congruence with a study conducted in southern Benin, where RO33 was the most frequent allele, both as monoclonal and polyclonal infections [47]. However, this is contrary to a study where RO33 was the least observed allele [48]. In Minna, in the North Central region of Nigeria, MAD20 was, however, the prevalent *msp1* allele family, while RO33 was the least frequent allele family [49]. Another study in North West Ethiopia showed MAD20 as the prevalent allele family [50]. However, caution should be exercised in generalizing this result, as our study was carried out in one state of the country in Nigeria. The overall MOI, which was high in our study, implies high transmission occurs in Nigeria, substantiating the WHO claim of the nation being one of the highest contributors to the global malaria burden [3]. In South Africa, malaria is a notifiable disease, and approaches like an operational and robust integrated malaria reporting system, the execution of proper and strict guidelines for malaria treatment, interborder collaborations, robust surveillance strategies and vector control strategies, etc. have led to a reduction in malaria to three out of nine provinces with low transmission [51,52]. 

Heterozygosity (*He*), is a measure of genetic diversity in polymorphic genes [1]. Generally, there was moderately high diversity in the studied parasite population. However, *msp1* gene diversity was unexpectedly higher in the South African isolates compared to the Nigerian isolates, while genetic diversity was similar in the *msp2* genes in the studied population. This observed pattern was consistent with the high MOI in the studied population between the countries. The high *He* observed in our study was also reported in studies conducted in Ethiopia [50], Tanzania, Malawi, Uganda, Burkina Faso and Sao Tome [53]. However, the unexpectedly high *He* observed in South Africa should not be seen, since this is a low transmission country where diversity would be expected to be low. This unexpected result shows that there are different clones or genotypes circulating in the population despite the low incidence of cases [1]. This could be due to the immigration of people from neighboring countries, leading to a diverse *P. falciparum* population in the South African isolates [52]. This was corroborated by Oyegoke et al., who found that Mozambique and Zimbabwe, with high transmission rates, shared the highest number of imported cases in South Africa [54]. *Msp1* gene diversity was high in Nigeria due to high transmission; this also shows different clones are in circulation. However, there is an advantage in high diversity in the population structure of *P. falciparum* regarding the evolution of drug resistance, and it is that the evolution of drug resistance is low or delayed in a diverse *P. falciparum* population, as dominant drug-resistant clones are liable to suppress and ultimately render extinct newly evolving drug-resistant clones through exploitative competition [55]. This possibly explains the reason why the evolution of drug resistance is low in sub-Saharan Africa, which has high malaria transmission rates, as compared to low transmission settings in Asia [55]. This phenomenon was substantiated by an epidemiological review of malaria intensity and drug resistance, where high chloroquine and chloroquine combination resistance was reported in very low transmission settings [56]. Contrastingly, high genetic diversity makes the development of effective vaccines difficult, as the reshuffling or recombination of varying genotypes would be high, thus decreasing the specificity and effectiveness of the malaria vaccine [57]. Notably, the geographical variations in the genetic diversity of the *P. falciparum* population due to the circulation of different clones in different locations is another limitation to developing effective malaria vaccines [58]. 

The most important observation of our study was the relationship between the allele RO33 and the age categories. We discovered that there was decrease in the RO33 allele as age increased. This could partly suggest that this variant is affected by natural immunity, because natural immunity to malaria develops with increases in age [59]. Thus, the significant decrease in RO33 with age in Nigeria could be owing to the formation of premunition in malaria-endemic areas and the maintenance of host immunity through continuous exposure to mosquito bites [60]. The decrease in the RO33 allele with increases in the age of participants suggests that a force could be responsible for the allelic suppression as the age increased in the studied area. This report is consistent with the study conducted in a rural setting of Cote d’Ivoire, where the MOI also decreased with an increase in age [61]. The reduction in RO33 in the study region could be due to the primary function of adaptive immunity. For instance, some studies suggested that antibodies against merozoite proteins could confer protection against clinical malaria [60], and the continuous exposure of individuals to malaria infection generates natural immunity against the disease [59]. Therefore, the decrease in RO33 allele with an increase in age could suggest the production of antibodies against RO33-like variants of *msp1* antigens, thereby providing protection against clinical malaria in the study area, as this finding was also observed in a study in Papua New Guinea, where antibodies against 3D7-like variants of *msp2* conferred protection against clinical malaria [49]. An adaptive immune system, also called acquired immunity, is an immunological response that is specific to pathogens and develops immunological memory, which rapidly eradicates the same pathogen in cases of subsequent infection [61]. This is evident since, as children grow into adolescents and adults, individuals are less likely to develop clinical malaria despite carrying the parasite, due to their natural immunity [59]. Furthermore, another possibility for the RO33 allele reduction is that the reactive oxygen species (ROS) created during respiratory bursts are toxic, damaging the merozoites close to the monocytes and thereby reducing the *msp1* protein [62]. Due to high transmission and consistent infections in the host, the immune system has, over time, built a memory which continuously fights *P. falciparum* infection as the host age increases. This phenomenon explains the protective effect of high *P. falciparum* genetic diversity in the host against malaria susceptibility [55]. However, this observation would require further experimental and immunological validation, as this was observed in a limited population. This study was not without limitations; the major limitation in this study was the small sample size, particularly in Nigeria, which makes it difficult to generalize the results. Moreover, though the *msp1* and *msp2* genes are highly polymorphic, fragment sizes may not reveal the entirety of the genetic diversity of *P. falciparum* in the studied population. In addition, data related to the vector were not captured during the sampling and, therefore, could not speak to transmission from the entomological perspective in selected sites. 

## 5. Conclusions

This study reported a higher MOI in the *P. falciparum msp1* gene in Nigeria compared to the South African isolates. There was high *msp1* gene diversity in South Africa, which could be due to imported cases. Despite the low incidence of cases reported in South Africa, the MOI was relatively high, suggesting high multiclonal infections and signifying active genetic recombination in the parasite population, leading to sustained genetic diversity of *P. falciparum* in the host. Therefore, caution should be exercised to avoid disease outbreaks. The RO33 allele reduction with an increase in age showed that the parasite population in the study area is under the influence of adaptive immunity in the presence of perennial transmission. These data provide the basis for understanding parasite population structure and transmission level, which are indices needed for effective malaria control strategies for malaria elimination.

## Figures and Tables

**Table 1:** Sequences of the primers for *msp1* and *msp2* genes in *P. falciparum* isolates.

Gene	Primers	Primer Sequences (5′→3′)	Annealing Temperature (°C)	Reference
Primary PCR				
*msp1*	M1-OR	5′-CTA GAA GCT TTA GAA GAT GCA GTA TTG-3′	54.2	[36]
	M1-OF	5′-CTT AAA TAG TAT TCT AAT TCA AGT GGA TCA-3′		
*msp2*	M2R	5′-ATG AAG GTA ATT AAA ACA TTG TCT ATT ATA-3′	53.2	[36]
	M2F	5′-CTT TGT TAC CAT CGG TAC ATT CTT-3′		
SECONDARY PCR				
K1	M1K1R	5′-AAA TGA AGA AGA AAT TAC TAC AAA AGG TGC-3′	58.2	[36]
	M1K1F	5′-GCT TGC ATC AGC TGG AGG GCT TGC ACC AGA-3′		
MAD20	M1MAD20R	5′-AAA TGA AGG AAC AAG TGG AAC AGC TGT TAC-3′	60.9	[36]
	M1MAD20F	5′-ATC TGA AGG ATT TGT ACG TCT TGA ATT ACC-3′		
RO33	M1RO33R	5′-TAA AGG ATG GAG CAA ATA CTC AAG TTG TTG-3′	62.2	[36]
	M1RO33F	5′-CAT CTG AAG GAT TTG CAG CAC CTG GAG ATC-3′		
IC3D7	M2ICR	5′-AAT ACT AAG AGT GTA GGT GCA TATGCT CCA-3′	62.7	[36]
	M2ICF	5′-TTT TAT TTG GTG CAT TGC CAG AAC TTG AAC-3′		
FC27	M2FCR	5′-AGA AGT ATG GCA GAA AGT AAC CCT TCT ACT-3′	61.6	[36]
	M2FCF	5′-GAT TGT AAT TCG GGG GAT TCA GTT TGT TCG-3′		

**Table 2:** Allelic proportion of *P. falciparum* in *msp1* and *msp2* genes between the two countries.

		South Africa				Nigeria			
Gene		TOTAL n (%)	Female	Male	*p*-Value	TOTAL n (%)	Female	Male	*p*-Value
			n (%)	n (%)			n (%)	n (%)	
*msp1*	K1	31 (39.7)	20 (25.6)	11 (14.1)	0.744	29 (56.9)	19 (37.3)	10 (19.6)	0.889
	RO33	6 (7.7)	4 (5.1)	2 (2.6)	0.685	46 (90.2)	29 (56.9)	17 (33.3)	0.451
	MAD20	12 (15.1)	7 (9.0)	5 (6.4)	0.506	15 (29.4)	9 (17.6)	6 (11.8)	0.650
	MAD20 + K1	8 (10.3)	5 (6.4)	3 (3.8)	0.792	9 (17.6)	5 (9.8)	4 (7.8)	0.527
	MAD20 + RO33	2 (2.6)	2 (2.6)	-	0.311	14 (27.5)	8 (15.7)	6 (11.8)	0.487
	RO33 + K1	1 (1.3)	1 (1.3)	-	0.477	26 (51.0)	17 (33.3)	9 (17.6)	0.918
	RO33 + MAD20 + K1	1 (1.3)	1 (1.3)	-	0.477	8 (15.7)	4 (7.8)	4 (7.8)	0.343
*msp2*	FC27	35 (44.9)	20 (25.6)	15 (19.2)	0.107	31 (60.8)	20 (39.2)	11 (21.6)	0.972
	IC3D7	34 (43.6)	22 (28.2)	12 (15.4)	0.747	10 (19.6)	7 (13.7)	3 (5.9)	0.696
	FC27 + IC3D7	14 (17.9)	9 (11.5)	5 (6.4)	0.835	10 (19.6)	7 (13.7)	3 (5.9)	0.696

*p*-value less than 0.05 was significant.

**Table 3:** Allelic distribution of *P*. *falciparum* in *msp1* and *msp2* among age categories.

Gene		Age Category						*p*-Value
		<19		20–40		40+		
		n	%	n	%	n	%	
*msp1*	K1	20	34.5%	27	46.6%	11	19.0%	0.571
	RO33	28	54.9%	16	31.4%	7	13.7%	0.001 *
	MAD 20	12	44.4%	10	37.0%	5	18.5%	0.173
	MAD 20 + K1	5	29.4%	9	52.9%	3	17.6%	0.909
	MAD 20 + RO33	10	62.5%	6	37.5%	0	0.0%	0.004 *
	RO33 + K1	15	57.7%	9	34.6%	2	7.7%	0.002 *
	RO33 + MAD 20 + K1	4	44.4%	5	55.6%	0	0.0%	0.243
*msp2*	FC	29	44.6%	23	35.4%	13	20.0%	0.001 *
	IC	13	29.5%	25	56.8%	6	13.6%	0.256
	FC+IC	12	50.0%	8	33.3%	4	16.7%	0.057

* Significance less than 0.05.

**Table 4:** Multiplicity of infection in *msp1* and *msp2* of *P. falciparum* from South Africa and Nigeria.

		South Africa	Nigeria	Independent *t*-Test *p*-Value
*Msp1*	MOI	1.24	1.63	<0.001 *
*Msp2*	MOI	1.20	1.24	0.213
Overall	MOI	2.44	2.87	<0.000 *
*Msp1*	*He*	0.81	0.54	
*Msp2*	*He*	0.61	0.59	

* *p*-value less than 0.05 was significant.

## Data Availability

Data are available upon request. The authors confirm that the data supporting this article are available within the manuscript. Raw data supporting the findings of this research are available upon request.

## Supplementary Materials

The following supporting information can be downloaded at: https://www.mdpi.com/article/10.3390/pathogens13020172/s1, Figure S1: Representative gel picture showing the msp1 and msp2 allele families MW: molecular weight, PC: positive control, NC: negative control, 1, 2, 3, 4, 5, 6, 7, 8 and 9, represent each well containing isolates.
