# Supplementary material for: A Comparative Study of Genetic Diversity and Multiplicity of Infection in Uncomplicated Plasmodium falciparum Infections in Selected Regions of Pre-Elimination and High Transmission Settings Using MSP1 and MSP2 Genes
Source: Pathogens. 2024 Feb 13;13(2):172. doi: 10.3390/pathogens13020172 (PMC10891941; doi:10.3390/pathogens13020172)
Supplement: Supplementary file 1 [file pathogens-13-00172-s001.zip › pathogens-2747944-supplementary.pdf]

Supplementary:

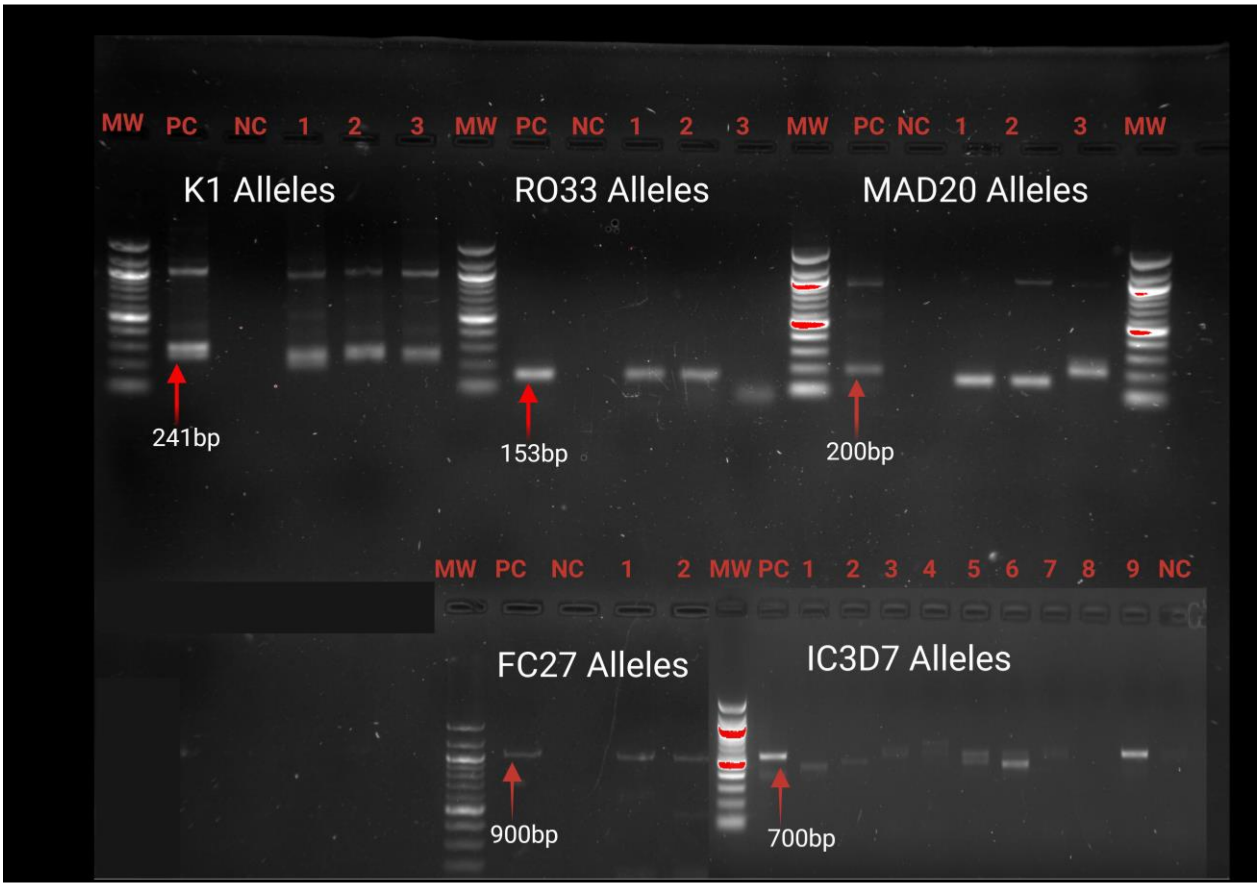

Supplementary Figure S1: Representative gel picture showing the *msp1* and *msp2* allele families MW: molecular weight, PC: positive control, NC: negative control, 1, 2, 3, 4, 5, 6, 7, 8 and 9, represent each well containing isolates.
